# Tadpoles respond to background colour under threat

**DOI:** 10.1038/s41598-018-22315-8

**Published:** 2018-03-06

**Authors:** Paula Cabral Eterovick, Izabela Santos Mendes, Jéssica Stephanie Kloh, Luan Tavares Pinheiro, Amadeus Bicalho Horta Portela Václav, Thiago Santos, Ana Sofia Buza Gontijo

**Affiliations:** Programa de Pós Graduação em Biologia de Vertebrados, Pontifícia Universidade Católica de Minas Gerais, 30535–610 Belo Horizonte, Brazil

## Abstract

The ability to respond to background colour is an important feature of species that might benefit from background matching camouflage. Tadpole colour patterns vary and could be associated with several functions, including defense. Because tadpoles are exposed to a wide array of visually oriented predators, they represent good models to study defensive colouration and associated behaviours. We tested whether a potentially disruptively camouflaged tadpole with a dark body crossed by yellow bars (*Ololygon machadoi*) is able to respond differently to matching light and dark natural background colours and an artificial blue contrasting background. We used a syntopic contrasting black tadpole (*Bokermannohyla martinsi*) as a control, expecting it not to respond to background colour in search for camouflage. *Ololygon machadoi* tadpoles chose light over blue backgrounds under threat, as expected, however they did not show preferential use of dark vs. blue backgrounds. *Bokermannohyla martinsi* did not respond to any combination of background colours. Our results suggest that *O*. *machadoi* tadpoles are able to respond to background colour, and may favor matching backgrounds under some circumstances. The potentially disruptive colouration of *O*. *machadoi* tadpoles may increase their repertoire of escape strategies, background matching being one of the options to escape predation.

## Introduction

Prey species that benefit from camouflage may be able to choose backgrounds that increase concealment^1,2^. Background colour, pattern, and complexity have been shown to play a role in prey detectability^3–5^. Both invertebrates and vertebrates show the ability to identify and choose backgrounds that match their body colours and such behaviour is expressed more intensely under conditions assessed as threatening by the prey^6–8^.

Amphibians show an amazing repertoire of colourations considered as defensive^9^, but there is limited information on their effectiveness against predators (maybe with the exception of aposematic colouration^10,11^) and the associated behavioural adaptations that may optimize survivorship of the prey^11,12^. Anuran tadpoles also show an impressive variety of colour patterns that could be associated with functions as diverse as predator defense and social interactions^13^. Tadpoles are vulnerable to a broad range of visually oriented predators such as Odonata, Belostomatidae, and vertebrates^5,14^, and most anuran species suffer high predation pressures during the larval stage^15^. Thus, tadpoles could benefit from predator avoidance caused by defensive colouration^13^ and are interesting models to study the defensive properties of such colourations against predators. They are also good models to study prey behaviours that maximize protection via reduced detection or avoidance by predators, because they are small and relatively easy to manipulate under experimental conditions^16^.

However, knowledge on the functions of tadpole colourations is incipient compared to other organisms such as birds and fishes^13^. For instance, there is a scarcity of quantitative data on predator avoidance via decreased detection probability promoted by tadpole colouration^13^. Both background matching and disruptive colouration (when animals have bars, stripes or patches that create the appearance of false edges, hiding the actual body outline) could play a role in avoiding predation^12,17,18^. However, for such colouration to function as defensive strategy, the prey should be able to choose the appropriate matching backgrounds^19^, and colour recognition may be key to this ability^8^. Otherwise, colour patterns may result from natural selection dependent upon the animal’s natural history, with patterns that provide better concealment in frequently used backgrounds under specific behavioural circumstances (such as hunting or escaping from predators) being selected for^20^.

**Figure 1:**
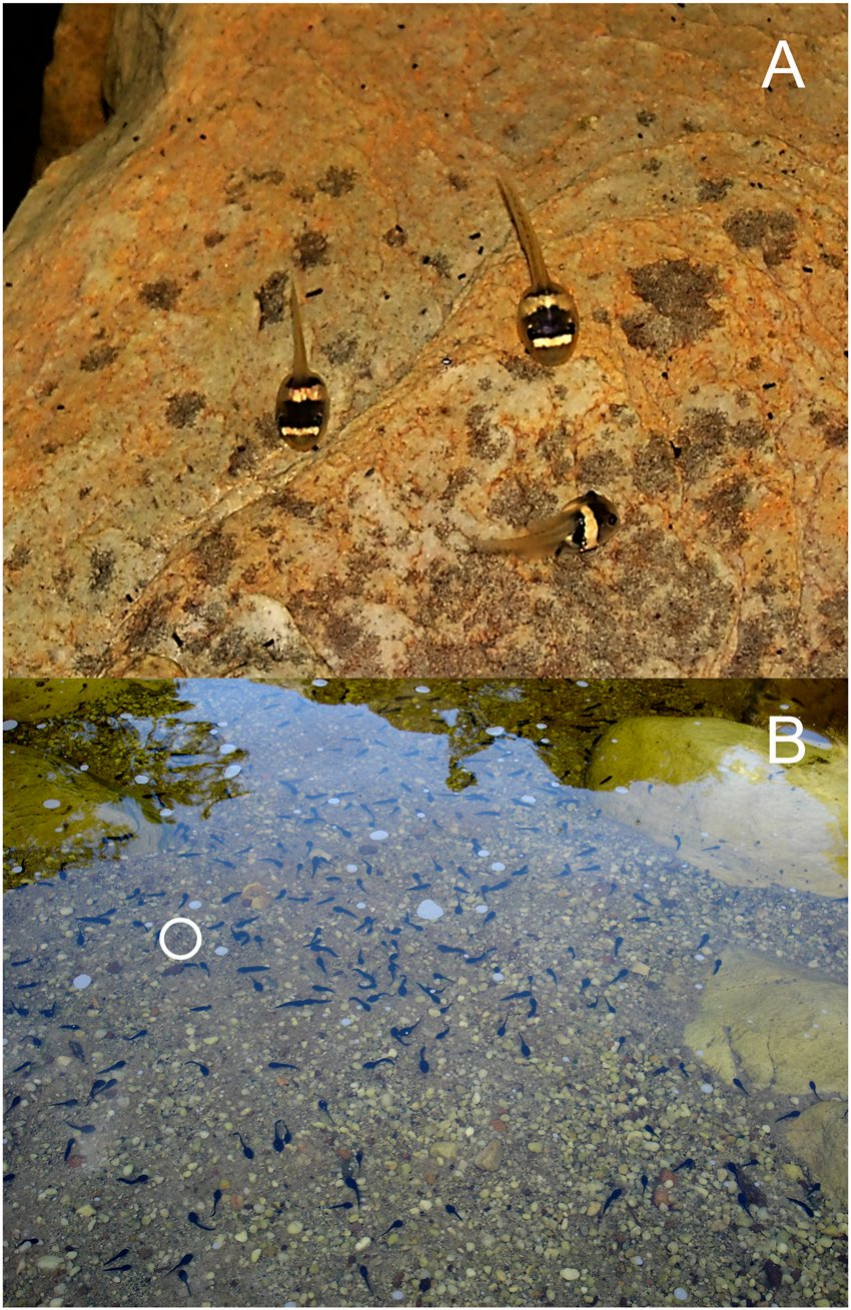
Tadpoles in their natural habitat. Tadpoles of (**A**) *Ololygon machadoi* and (**B**) *Bokermannohyla martinsi* on their natural backgrounds in a stream at the RPPN Santuário do Caraça, southeastern Brazil. A tadpole of *O*. *machadoi* is circled in (**B**) for comparison of background contrast level with *B*. *martinsi* tadpoles.

In order to test whether tadpoles are capable of actively positioning themselves on backgrounds where they look disruptive, we used *Ololygon machadoi* (Bokermann and Sazima, 1973) as a model. *Ololygon machadoi* tadpoles have a potentially disruptive colouration^18^, with a dark body crossed by bright yellow bars^21,22^ (Fig. 1A). In accordance with the definition of disruptive colouration^18^, the contrast between the dark and yellow parts of the tadpole body is intense, and they reach the outline of the body, interrupting it and blending different parts of the tadpole to different backgrounds in a way that hinders the detection of real body shape. Tadpoles are active during the day^21^. Their colours match debris deposits or rocks covered with periphyton or debris (dark) as well as exposed rocks (light, yellowish under the tanin-stained waters) at the stream beds where they use to rest at montane meadows in southeastern Brazil. Predation levels upon *O*. *machadoi* tadpoles by visually oriented aquatic insects (Odonata and Heteroptera) or birds did not differ in most cases between light and dark backgrounds under experimental conditions^5^. These tadpoles could be disruptive through differential blending^18^ on both light and dark backgrounds, and they used both in the same frequency when given a choice^5^. In this scenario, a lack of preference for any of these two background colours could mean (1) that both confer some protection to tadpoles or (2) that tadpoles don’t respond to background colour. However, a test of the tadpole’s preference for these backgrounds against a contrasting background remains to be done. By adding a blue background that is certainly contrasting to the whole tadpole body, these two hypotheses could be tested. Tadpoles should prefer either yellow or dark over blue backgrounds if they are able to choose backgrounds that match their bodies. Additionally, previous tests for background preference were conducted in the absence of aversive stimuli^4^, and threats perceived by tadpoles could influence their background choice towards more concealing backgrounds^7^.

*Bokermannohyla martinsi* is the only other syntopic species whose tadpoles are as abundant as those of *O*. *machadoi* at the studied area along the whole year, and these two species would be the most abundant prey to potential tadpole predators. Being syntopic, these tadpoles are exposed to the same habitat and background colours, what makes them comparable to *O*. *machadoi* tadpoles regarding any experience based influence of visual perception of their surroundings. *Bokermannohyla martinsi* tadpoles position themselves on rocks most of the time^23,24^. The whitish stream bed rocks look yellow from outside and are very contrasting with *B*. *martinsi* tadpoles (Fig. 1B). Considering this and the high activity levels of these tadpoles, even in the presence of predator cues (G. Melo and PCE, unpubl. data), we assumed that camouflage is unlikely to be a defensive strategy for them. Thus, we used tadpoles of *Bokermannohyla martinsi* as a control to be compared with *O*. *machadoi* tadpoles because (1) they occur in the same streams and are exposed to the same backgrounds as *O*. *machadoi*, (2) they are unlikely to use camouflage for defense. We did not expect them to search for concealing backgrounds, probably choosing their backgrounds based on needs other than camouflage (e.g., thermoregulation, food availability). We thus expected *B*. *martinsi* tadpoles to use our experimental background colours randomly because they did not differ in any aspect other than colour. On the other hand, we expected *O*. *machadoi* to choose either light or dark backgrounds instead of a contrasting blue background, especially when under threat.

Under light conditions, porphyropsin is the predominant retinal pigment in tadpole eyes whereas most of this pigment is replaced by rhodopsin under dark conditions^25^. Tadpoles maintained in the dark under experimental conditions responded with an increase in porphyropsin under either blue or yellow-green lights, but not under red lights. Tadpoles can be thus considered relatively insensitive to red light compared to blue or yellow-green spectra in a way that the exposure to only red light would produce a retinal response like that produced in the dark^25^. For these reasons, we conducted our experiments within the blue, yellow-green spectra where tadpole eyes could be expected to have colour perceptions like those of human eyes, that is, easier for us to interpret. We hypothesized that a preference for either yellow or dark backgrounds over blue backgrounds in *O*. *machadoi* but not in *B*. *martinsi* tadpoles would show that tadpoles of *O*. *machadoi* can see background colour and respond to it in a way to maximize its concealment.

## Methods

The study was conducted at the Reserva Particular do Patrimônio Natural (RPPN) Santuário do Caraça (20°05′S, 43°29′W; 750–2,072 m alt.). This private reserve encompasses 12,403 ha in the southern portion of the Espinhaço Mountain Range, southeastern Brazil. The climate has a rainy season from October to March and a dry season from April to September, with mean monthly temperatures varying from 13 to 29 °C^26^. The reserve has several permanent streams where tadpoles of *Bokermannohyla martinsi* (Bokermann, 1964) and *Ololygon machadoi* are the most abundant and can be found year-round^27,28^.

**Figure 2:**
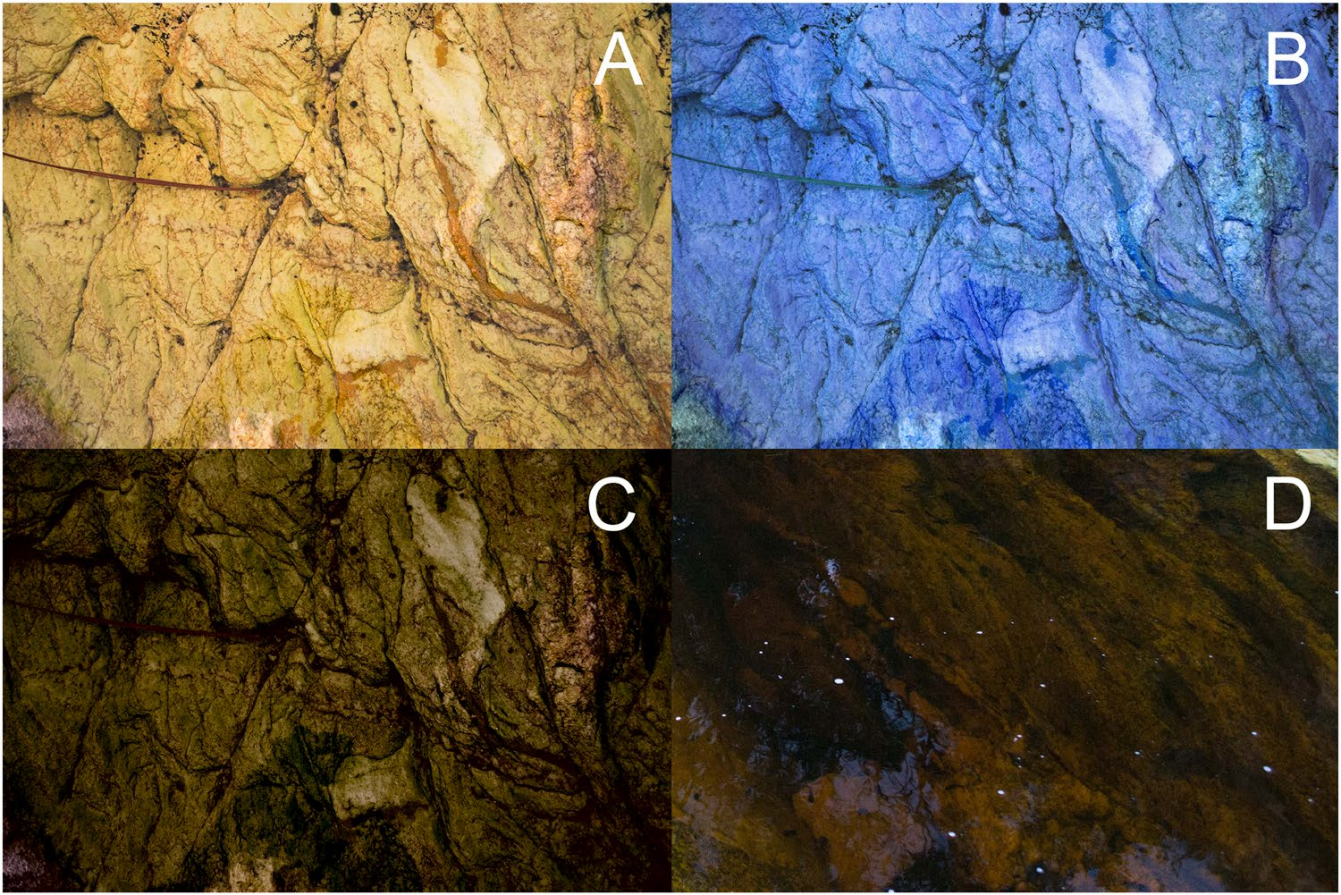
Pictures used in tadpole background choice experiments. These included a picture of a natural light stream background section (**A**), the same picture digitally manipulated to an artificial blue tone contrasting with all tadpole body colours (**B**) and to darker shades (**C**) corresponding to dark sections of natural stream backgrounds. An example of a real dark section of stream background is shown in (**D**).

Background features other than colour may also influence tadpole choice^5,24^, thus we prepared experimental backgrounds to test tadpoles for background choice based solely on colour detection. The yellow background was a picture of an actual stream bed light background (Fig. 2A). The blue background (Fig. 2B) consisted in the same picture digitally manipulated to resemble the colour of an artificial blue background intended to make the tadpoles easily detectable. We chose blue because tadpoles are sensitive to this colour and it is far from the yellow spectrum of natural backgrounds and tadpole bodies. The dark background (Fig. 2C) was also made from the same picture digitally manipulated to resemble the colours of a real dark stream background (Fig. 2D). We considered as dark backgrounds that were actually dark and not just more shaded. We made all experimental backgrounds based on the same picture to standardize texture because visual complexity is also likely to influence background choice for improved concealment^1^. We used Adobe Photoshop CS6, version 13.0 × 64 (Adobe Photoshop 1990–2012, Adobe Systems Incorporated) to modify the pictures in LAB colour mode and we quantified “L”, “a” and “b” components in the whole original and modified pictures (Table 1). LAB is a colourimetric method to quantify picture components, where L represents “lightness” and is critical for contrast and image spatial formation, “a” represents a position in the gradient between red and green, whereas “b” represents a position in the gradient between blue and yellow^29^. The “a” component was relatively constant in all pictures used in the experiment, the “b” component was modified in the blue picture (we moved the position in the gradient towards blue), and we decreased the “L” component in the dark picture (Table 1). We printed the pictures using a HP Colour LaserJet 2600n with 600dpi resolution and automatic calibration.

**Table 1:** LAB colour measurements (L, a, b) of background pictures used in experiments (except for natural dark backgrounds that were just used for colour reference) to test whether *O*. *machadoi* and *B*. *martinsi* tadpoles respond to background colour in the absence of predator cues and after an aversive stimulus. *Manipulated backgrounds.

LAB channel	Background colours			
	Yellow	Blue*	Dark*	Dark
L	165.7 ± 36.1	131.5 ± 30.1	41.9 ± 32.4	37.4 ± 18.6
a	137.4 ± 4.6	130.8 ± 5.1	132.5 ± 3.5	132.0 ± 3.3
b	168.8 ± 9.9	87.3 ± 10.4	169.5 ± 10.5	140.9 ± 9.0

Although printing quality looked good to us, some deviation from target colours is likely to happen during printing. Thus, we compared reflectance of tadpole’s yellow and dark body parts with reflectance of printed yellow and dark backgrounds respectively, and also measured reflectance of rocks from the stream bottom. We investigated the correspondence among tadpole, natural background and picture considering different visual systems of the species involved not only in this experiment, but also in the evolution of background colour choice as a defensive behaviour in *O*. *machadoi*. We used a spectrophotometer USB2000 + UV-VIS-ES (Ocean Optics Inc.) calibrated between 250 and 750 nm with a deuterium/tungsten light source. We used Spectralon ® (WS-1-SL, Ocean Optics Inc) as a white control for calibration. Reflectance measurements were obtained with an optic fiber R400-7-UV-VIS (Ocean Optics Inc.) and a source of pulsed xenon light (PX-2, 220 Hz, 220–750 nm, Ocean Optics Inc.). We mounted the light receptor on a black support (RPH-1, Ocean Optics Inc.) to allow the standardization of an angle of 45° and a distance of 0.5 cm from the object to me measured. Spectral curves were visualized in the software SpectraSuite (Ocean Optics Inc.). The reflectance peaks were coincident except for UV wavelengths reflected on the yellow bars on tadpoles’ bodies. The yellow bars had two reflectance peaks, corresponding to yellow and UV spectra (I. M. Martins, A. Vasconcelos, T. Motta and P. C. Eterovick, unpubl. data). Natural stream backgrounds do not reflect UV either, thus we believe the pictures represented them properly and matched tadpole colours based on the vision of both the tadpoles and potential predators which see UV such as birds^30^. Experiments on bird predation upon *O*. *machadoi* tadpoles on the same pictures used in this study are showing that tadpoles are actually more visible on blue backgrounds (as we first assumed) compared to yellow and dark, and this is likely dependent on the amount of incident UV (I. M. Martins, A. Vasconcelos, T. Motta and P. C. Eterovick, unpubl. data). However, under natural conditions, little or no UV is expected to reach the stream bottom through tanin stained waters (J. A. Endler, pers. comm.).

We taped printed laminated pictures of yellow, dark and blue backgrounds to the bottom of six plastic trays (43 cm length, 30 cm width, 9 cm height). These backgrounds were combined in pairs (each colour covering half the tray bottom) resulting in three treatments: dark/yellow, blue/yellow, and blue/dark (two trays for each treatment). We tested a total of 108 tadpoles of each species per treatment, summing a total of 324 tadpoles of *B*. *martinsi* and 324 tadpoles of *O*. *machadoi* tested during three consecutive days (6–8 October 2015) with similar weather conditions. We conducted 54 runs of the experiment. Each run consisted of a simultaneous test of six tadpoles (two per treatment) of *O*. *machadoi* followed by a simultaneous test of six tadpoles of *B*. *martinsi*. Each tadpole was tested only once. The experimental procedures were approved by the ICMBio/Sisbio (45302–1, 45302–2) and the Ethics Comitee of the Pontifícia Universidade Católica de Minas Gerais (CEUA). All experiments were performed in accordance with relevant guidelines and regulations.

In order to minimize stress to tadpoles, we collected them in groups of 36 tadpoles of *B*. *martinsi* and 36 tadpoles of *O*. *machadoi* from a third order stream^31^ (20°06′40”S, 43°28′48″W; 1254 m alt.) and kept them in styrofoam boxes with stream water for two hours at most for each set of six runs of the experiment. We filled each tray with 2.5 l of stream water and placed them lined (alternating treatments) on a shaded platform about 50 m from the stream. We placed one tadpole in the middle of each tray, moved away and waited 3 min for it to position itself somewhere in the tray. We observed that tadpoles were sometimes more active when they entered the tray, but after 3 min they were already presenting an activity level comparable to their natural behaviour in the stream and did not change further. Just one person remained next to the platform and close enough to see where in the trays tadpoles were. At 3 min we recorded the background colour where each tadpole was positioned and the person who was close to the trays inserted a wood stick vertically towards the tadpole’s body slowly and with the same approaching speed until it touched the tadpole or elicited a escape response. This procedure has already been used as an aversive stimulus to elicit escape responses in tadpoles^24^. Trays on the platform stood at about waist level of the person who moved slowly from tray to tray, avoiding disturbance to tadpoles before they were tested. We then recorded the background colour where the tadpole stopped after being disturbed. After completing the trials with six tadpoles of one species, we did the same experiment with the other species, and then we re-started the sequence until we had done it six times for each species. After that, we went back to the stream, released the tested tadpoles downstream from sampling points and collected new tadpoles for another block of tests. We changed the water in the trays for new fresh stream water immediately before every trial to avoid the possible influence of any alarm substance secreted by threatened tadpoles in the behaviour of the next individuals to be tested. We collected all the water used in the experiments at the same point of the stream and discarded it downstream afterwards.

We wanted to know whether any colour would be preferred by tadpoles before the aversive stimulus, and how tadpoles would react to the aversive stimulus based on the colour it was on initially (we considered two alternative behaviours: choose the same colour again or change colour). For each treatment (dark/yellow, blue/yellow, and blue/dark) we had four possible situations given by the combinations of each colour chosen initially and the two alternative behaviours. We used the dark/yellow treatment (simulating natural background colours the tadpoles are naturally exposed to) to test whether any of the species would show preference for either of these colours. Then, in the treatments blue/yellow and blue/dark, we evaluated whether tadpoles would prefer familiar colours against an unfamiliar contrasting colour. We expected *O*. *machadoi* to favor either yellow, dark or both these backgrounds against blue backgrounds, and *B*. *martinsi* to show no preference for any background colour. We also expected *O*. *machadoi* to be more willing to move to its preferred backgrounds after disturbance or remain on them. We compared the numbers of tadpoles on the two colours of each treatment (initial colour chosen by the tadpole) and the numbers choosing the same or the alternative colour (behaviour) after disturbance with analyses of variance. We used each run of the experiment (with six tadpoles of each species) as sample units. We conducted these analyses for each treatment and each species separately in R^32^. We adopted the significance level of *P* ≤ 0.05.

## Results

Tadpoles of both species responded to the aversive stimulus in varied ways. Some moved very short distances (a few centimeters), others moved across the tray once, twice or several times, some remained in the same half of the tray, some moved to the opposite half, some moved to the opposite half and back one or more times. *Bokermannohyla martinsi* tadpoles usually moved more and covered longer distances before stopping when compared to *O*. *machadoi* tadpoles (although we did not measure it quantitatively).

**Figure 3:**
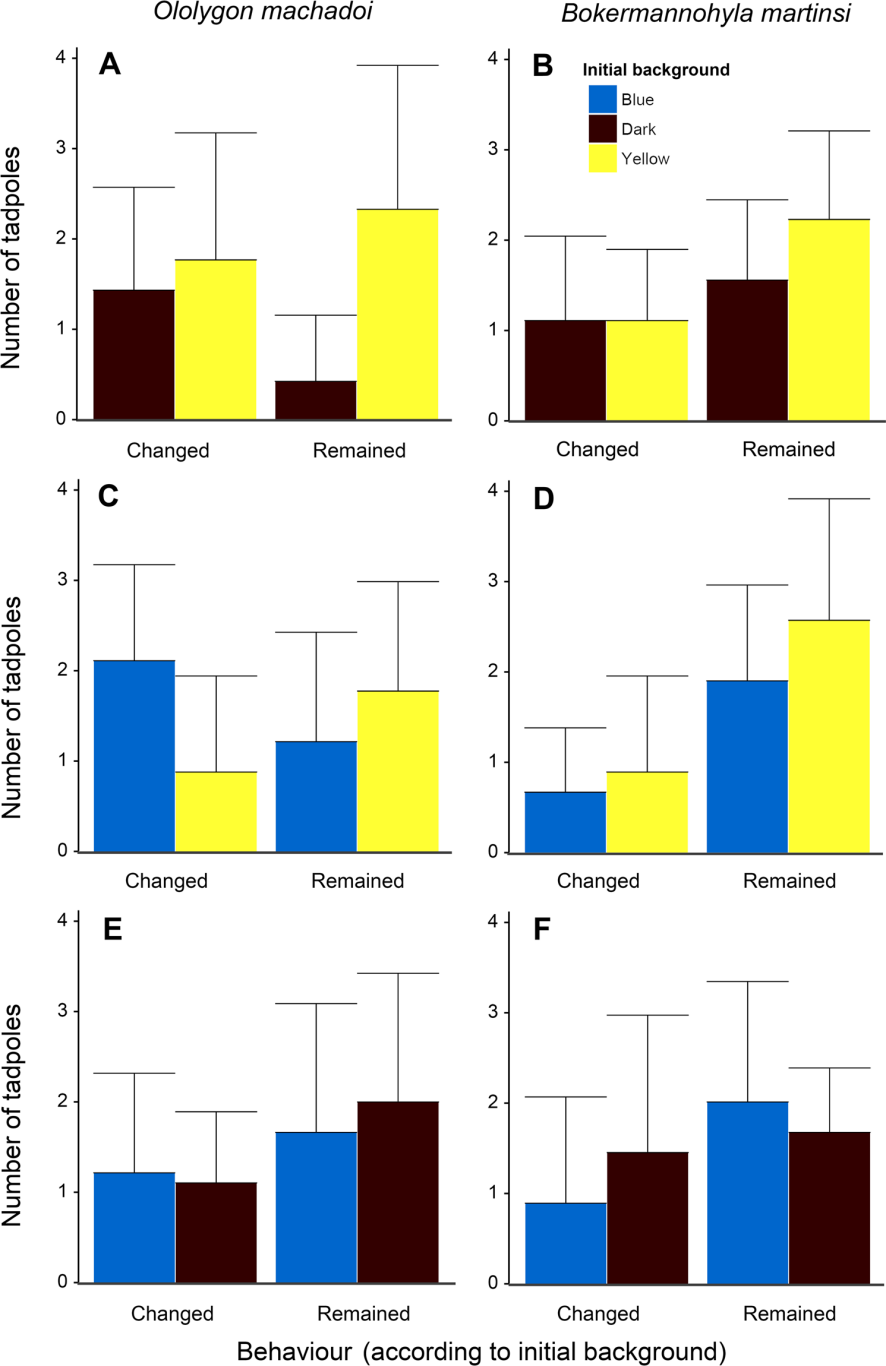
Background colour choice and behaviour under threat. Mean number of tadpoles of *Ololygon machadoi* (**A**,**C**,**E**) and *Bokermannohyla martinsi* (**B**,**D**,**F**) that used the same initially chosen colour (Remained) or changed background colour (Changed) after being disturbed in each of three treatments (given by combinations of two colours): dark/yellow (**A**,**B**), blue/yellow (**C**,**D**), blue/dark (**E**,**F**). Bars represent SDs.

In the treatment with yellow/dark backgrounds, *O*. *machadoi* tadpoles preferred yellow over dark backgrounds initially (initial background: MS = 11.11, F = 7.11, df = 32, p = 0.012; Fig. 3A: yellow vs. black bars). The total frequency of the two behaviours did not differ, but tadpoles initially on yellow backgrounds had a nearly significant tendency to choose the same colour after disturbance compared to tadpoles initially on dark backgrounds (interaction: MS = 5.44, F = 3.48, df = 32, p = 0.071; Fig. 3A: “Remained”). In the treatment with yellow/blue backgrounds, *O*. *machadoi* tadpoles that chose yellow backgrounds initially were more likely to choose them again after disturbance whereas tadpoles that chose blue backgrounds initially were more likely to change to yellow backgrounds after disturbance (interaction: MS = 7.11, F = 5.57, df = 32, p = 0.025; Fig. 3B: “Changed”). In the treatment with blue/dark backgrounds, tadpoles did not choose any colour initially or favor any behaviour in general or depending on initial colour.

*Bokermannohyla martinsi* tadpoles seemed to choose colours randomly, as expected (no significant results for initial colour or the interaction between initial colour and behaviour in any treatment). They chose the same colour again after being disturbed more frequently than changed colour in the dark/yellow (behaviour: MS = 5.44, F = 6.82, df = 32, p = 0.014) and blue/yellow (behaviour: MS = 18.78, F = 16.69, df = 32, p = 0.0003) treatments but not in the blue/dark treatment.

## Discussion

Ololygon machadoi tadpoles used yellow backgrounds preferentially compared to dark backgrounds before an imposed aversive stimulus, possibly indicating an innate (or learnt) preference. They were also likely to remain on yellow backgrounds or move towards them after being exposed to an aversive stimulus in dark/yellow treatments. When exposed to blue/yellow backgrounds, tadpoles were not hesitant to explore the unfamiliar blue background initially, but once disturbed on blue backgrounds most of them promptly moved to yellow backgrounds. In this case, the defensive behaviour of concealing background choice could have been expressed under predation risk, as it was observed for the killifish (*Heterandria formosa*^1^). On the other hand, *B*. *martinsi* tadpoles, used as a control, did not show any significant change in use of background colours before or after the aversive stimulus in any treatment, as expected. After disturbance, most *B*. *martinsi* tadpoles positioned themselves on the same colour chosen initially in two of the three treatments (dark/yellow and blue/yellow), what may have been a fortuitous consequence of the higher activity levels of this species. However, there were no interactions between this behaviour and colour, different from *O*. *machadoi*.

Since we controlled for background texture and patchiness, it is reasonable to assume that *O*. *machadoi* tadpoles responded to background colour. Amphibian larvae can see in the yellow and blue spectra^25^, and maybe the blue backgrounds that do not occur in their habitats looked unfamiliar to them or was considered as unsafe, being thus avoided under threat. They were able to discriminate between concealing and non concealing backgrounds using, in this case, the “b” channel. Interestingly, yellow and dark backgrounds have similar hues (components “a” and “b”) but different lightness (component “L”), and tadpoles seemed to favor yellow over dark backgrounds. That means tadpoles were also able to respond to changes in lightness (“L”).

If our manipulation before the experiments caused some stress to tadpoles, the choice of yellow over dark backgrounds would have been expected under a perceived risk also in the beginning of the dark/yellow treatment, reinforcing our results. However, we believe this is unlikely because we were careful to wait for tadpoles to achieve normal activity levels (as observed in natural habitats) in the trays before we started the experiments. Thus, the preference for yellow over dark backgrounds by *O*. *machadoi* tadpoles before the imposed aversive stimulus could be influenced by reasons other than protection. Because of the tannin-stained water in the streams at the study site, shallow and warmer areas of the stream usually look lighter, so maybe tadpoles use lightness as a cue to warmer microhabitats to thermoregulate. It’s been shown that tadpoles are able to select microhabitats based on suitable temperatures^33^. However, in our experiments temperatures did not vary because we used water collected at the same point in the stream immediately before each trial.

We expected a significant decrease in use of blue backgrounds after the aversive stimulus also in the blue/dark treatment, which did not happen. In the natural habitat, *O*. *machadoi* tadpoles, when disturbed, move short distances and then stop motionless (PCE, pers. obs). Reduced movement is a known strategy to reduce predation risk by tadpoles^34^. Thus escaping tadpoles would face a trade-off between two strategies: moving as little as possible and moving enough to reach the most concealing background. For some reason, tadpoles in the blue/black treatment may have favored the first strategy (although we did not notice differences in distance covered among treatments, we did not measure it quantitatively). If tadpoles really associate yellow backgrounds with warmer temperatures, stopping motionless would be a good strategy once in microhabitats perceived as cooler. Tadpoles are capable of adjusting their response to predators based on perceived risk and context^35^. Although the experimental conditions were the same for tadpoles in all treatments, individuals may have responded differently leading to an unexpected pattern of colour choice in the blue/dark treatment. Patterns of risk experienced by prey over time can influence escape behaviour and decision making^35^, and recent experiences are likely to have a greater influence in response to predation risk^36^.

Our results indicate that *Ololygon machadoi* tadpoles are sensitive to background colour, what is in accordance with their potentially disruptive colouration. In disruptive animals, although internal contrast among colours (i.e., adjacent patches of the animal) should be high enough to be more visible than real body outline, matching of one or more body colours with the background is important to increase concealment and protection^18^. However, their repertoire of escape strategies may include other escape mechanisms such as fleeing or decreased mobility. These tadpoles are threatened by several visually oriented predators in their natural habitats^5^, so they may be able to adjust escape strategy according to perceived risk, or they may even respond randomly to avoid predator learning. It is also possible that the background where tadpoles are when threatened will influence their response, for instance, decreasing mobility in more contrasting backgrounds^34^. It is likely that the potentially disruptive colour pattern of *O*. *machadoi* tadpoles allow them to diversify their repertoire of escape strategies and backgrounds used to escape (i.e., yellow or dark). Birds have been shown to prey less on targets with disruptive patterns against a matching background as opposed to targets with a similar colour pattern that does not disrupt the object’s outline^17^. The colouration of *O*. *machadoi* could function as disruptive on dark and yellow backgrounds with differential blending (i.e., one colour blending to the background and the other standing out^18^), but not on blue ones, making its effectiveness against predation context dependent. Comparison of escape strategies among tadpoles with varied colour patterns will be interesting to further understand the role of tadpole defensive colourations in tropical habitats with a great array of potential predators.

### Data availability statement

The datasets generated and/or analysed during the current study are available from the corresponding author on reasonable request.
